# Heterozygous familial hypercholesterolaemia in a pair of identical twins: a case report and updated review

**DOI:** 10.1186/s12887-019-1474-y

**Published:** 2019-04-11

**Authors:** Noor Shafina Mohd Nor, Alyaa Mahmood Al-Khateeb, Yung-An Chua, Noor Alicezah Mohd Kasim, Hapizah Mohd Nawawi

**Affiliations:** 1 Institute for Pathology, Laboratory and Forensic Medicine (I-PPerForM), Universiti Teknologi MARA (UiTM), Sungai Buloh Campus, Jalan Hospital, 47000 Sungai Buloh, Selangor Malaysia; 2 Departments of Paediatric, Biochemistry and Chemical Pathology, Faculty of Medicine, Universiti Teknologi MARA (UiTM), 47000 Sungai Buloh, Selangor Malaysia

**Keywords:** Familial hypercholesterolaemia, *LDLR*, *ABCG8*, Premature atherosclerosis, Coronary artery disease

## Abstract

**Background:**

Familial hypercholesterolaemia (FH) is the most common inherited metabolic disease with an autosomal dominant mode of inheritance. It is characterised by raised serum levels of total cholesterol (TC) and low-density lipoprotein cholesterol (LDL-c), leading to premature coronary artery disease. Children with FH are subjected to early and enhanced atherosclerosis, leading to greater risk of coronary events, including premature coronary artery disease. To the best of our knowledge, this is the first report of a pair of monochorionic diamniotic identical twins with a diagnosis of heterozygous FH, resulting from mutations in both *LDLR* and *ABCG8* genes.

**Case presentation:**

This is a rare case of a pair of 8-year-old monochorionic diamniotic identical twin, who on family cascade screening were diagnosed as definite FH, according to the Dutch Lipid Clinic Criteria (DLCC) with a score of 10. There were no lipid stigmata noted. Baseline lipid profiles revealed severe hypercholesterolaemia, (TC = 10.5 mmol/L, 10.6 mmol/L; LDL-c = 8.8 mmol/L, 8.6 mmol/L respectively). Their father is the index case who initially presented with premature CAD, and subsequently diagnosed as FH. Family cascade screening identified clinical FH in other family members including their paternal grandfather who also had premature CAD, and another elder brother, aged 10 years. Genetic analysis by targeted next-generation sequencing using MiSeq platform (Illumina) was performed to detect mutations in *LDLR, APOB100, PCSK9, ABCG5, ABCG8, APOE* and *LDLRAP1* genes. Results revealed that the twin, their elder brother, father and grandfather are heterozygous for a missense mutation (c.530C > T) in *LDLR* that was previously reported as a pathogenic mutation. In addition, the twin has heterozygous *ABCG8* gene mutation (c.55G > C). Their eldest brother aged 12 years and their mother both had normal lipid profiles with absence of *LDLR gene* mutation.

**Conclusion:**

A rare case of Asian monochorionic diamniotic identical twin, with clinically diagnosed and molecularly confirmed heterozygous FH, due to *LDLR* and *ABCG8* gene mutations have been reported. Childhood FH may not present with the classical physical manifestations including the pathognomonic lipid stigmata as in adults. Therefore, childhood FH can be diagnosed early using a combination of clinical criteria and molecular analyses.

## Background

Familial hypercholesterolaemia (FH) is the most common inherited metabolic disease with an autosomal dominant mode of inheritance. It is characterised by raised serum levels of total cholesterol (TC) and low-density lipoprotein cholesterol (LDL-c), leading to premature coronary artery disease (CAD). Homozygous FH (HoFH) patients manifest a more severe clinical phenotype compared to heterozygous FH (HeFH). Globally in the community, the HeFH prevalence is reported to be 1:200–500, but more recent reports showed higher prevalence of 1:100–250 [1–5]. On the other hand, the prevalence of HoFH is much lower [6], although a greater prevalence is reported among certain populations, apparently due to founder effects [7].

Familial Hypercholesterolaemia is mainly caused by mutations in the low-density lipoprotein receptor (*LDLR*) gene that results in reduced uptake and clearance of LDL-c [8]. Less common causes of FH are mutations in the genes encoding apolipoprotein B-100 (*APO B-100*) and proprotein convertase subtilisin/kexin 9 (*PCSK9)*^9^. Rarely the autosomal recessive hypercholesterolemia (*ARH*) LDL receptor adaptor protein gene (*LDLRAP1*) can also result in a similar HoFH phenotype [9]. Over 1200 different types of mutations were described in the *LDLR* gene [10]. Few variants in *APOB-100* gene, affecting the LDL receptor-binding domain of apolipoprotein B were reported leading to a disorder that is called familial defective Apo B (FDB) [5, 11] and almost all appear within a relatively small coding region near p.3527 [12]. More than 30 *PCSK9* gain-of-function (GOF) mutations have been reported to be the cause of FH since the discovery of *PCSK9* gene in 2003 [11, 13].

Sitosterolemia is an extremely rare autosomal recessive disorder of sterol metabolism caused by mutations in one of two genes ATP-binding cassette (ABC) sub-family G members 5 and 8 (*ABCG5* and *ABCG8*) and it is characterised by increased absorption and decreased biliary excretion of plant sterols and cholesterol, resulting in elevated serum levels of plant sterols like sitosterol and campesterol [14, 15]. Subjects suffering from sitosterolemia also primarily develop xanthomas and even premature coronary atherosclerosis [16].

Genetic testing is helpful for confirmation of FH diagnosis. However, the causative mutation may remain undetected in certain patients, even after thorough screening for mutation using the currently available advance techniques [17]. Many mutations were ruled out as variants with uncertain significance probably due to lack of functional evidence, family co-segregation data or benign outcome from in silico analysis. However, there are evidences that accumulation of multiple small-effect common FH variants, including some autosomal recessive genes such as *ABCG5/8*, may still cause significant elevation of LDL-c, dubbed as polygenic FH [18, 19]. Lack of genetic finding among the suspected FH patients may also be caused by elevated LDL-c resulted from lifestyle factors and secondary causes (e.g. hypothyroidism or nephrotic syndrome), or a combination of these factors that might explain the clinical phenotype in terms of hypercholesterolemia or CAD with the absence of gene mutations [20].

The rapid advances in next generation sequencing (NGS)-based techniques have made them more accessible for diagnostic purposes. Most of the sequencing methods that are established to diagnose hereditary disorders usually concentrate on the exome, which constitutes about 1% of the whole human genome. Additionally, the computer-based prediction tools that are used to detect the pathogenicity of the gene sequence variants are now established and used to estimate the deleterious effect of the various amino acid changes [21].

There are potentially as many as 4.5 million individuals in Europe with HeFH and possibly 35 million around the world, of whom 20–25% of these are children and adolescents [5, 22]. Given the high prevalence of FH, it is estimated that one baby is born with FH every minute [22]. However, FH is very much under diagnosed and undertreated globally [5]. Children with FH have higher risk of early coronary events and death from myocardial infarction due to premature atherosclerosis. To the best of our knowledge, this is the first report of an Asian pair of monochorionic diamniotic identical twin with heterozygous FH caused by mutations in both *LDLR* and *ABCG8* FH candidate genes. Furthermore, to the best of our knowledge, this is the first study which utilised targeted next-generation sequencing (TNGS) technique to identify the causative gene mutations for FH among the Malaysian population.

## Case presentation

This is a rare case of a pair of 8-year-old monochorionic diamniotic identical twin, of Malaysian Indian descent, who on family cascade screening were diagnosed as definite FH, according to the Dutch Lipid Clinic Criteria (DLCC) with a score of 10. No lipid stigmata were identified. Baseline lipid profiles revealed severe hypercholesterolaemia, (TC = 10.5 mmol/L, 10.6 mmol/L; LDL-c = 8.8 mmol/L, 8.6 mmol/L respectively) (Table 1**).** Their father is the index case who initially presented with premature CAD, and subsequently diagnosed as FH. Family cascade screening identified clinical FH in other family members including their paternal grandfather who also had premature CAD, and another elder brother, aged 10 years.

**Table 1:** Summary of the biochemical and molecular findings among the family members

Family member	Clinical FH diagnosis	TC mmol/lL	TG mmol/L	HDL-C mmol/L	LDL-C mmol/L	dbSNP ID	Predicted effect
Proband	Definite FH	10.0	1	1.5	8.6	*LDLR*: rs121908026	Probably Damaging
Older brother (Hypercholesterolemic)	Probable FH	9.6	0.6	0.3	7	*LDLR*: rs121908026	Probably Damaging
Grandfather	Definite FH	9.3	1.4	1	7.7	*LDLR*: rs121908026	Probably Damaging
Twin, youngest brother	Definite FH	10.6	0.7	1.8	8.6	*LDLR*: rs121908026	Probably Damaging
						*ABCG8*: rs11887534	Possibly Damaging
Twin, youngest brother	Definite FH	10.8	0.6	1.7	8.8	*LDLR*: rs121908026	Probably Damaging
						*ABCG8*: rs11887534	Possibly Damaging
Eldest brother (Normolipaemic)	Possible FH	5.2	0.4	2.6	2.4	*LDLR*: rs2738442	Benign
						*ABCG8*: rs11887534	Possibly Damaging
Mother	Possible FH	5.3	0..9	2	2.8	*LDLR*: rs2738442	Benign
						*ABCG8*: rs11887534	Possibly Damaging

**Fig. 1:**
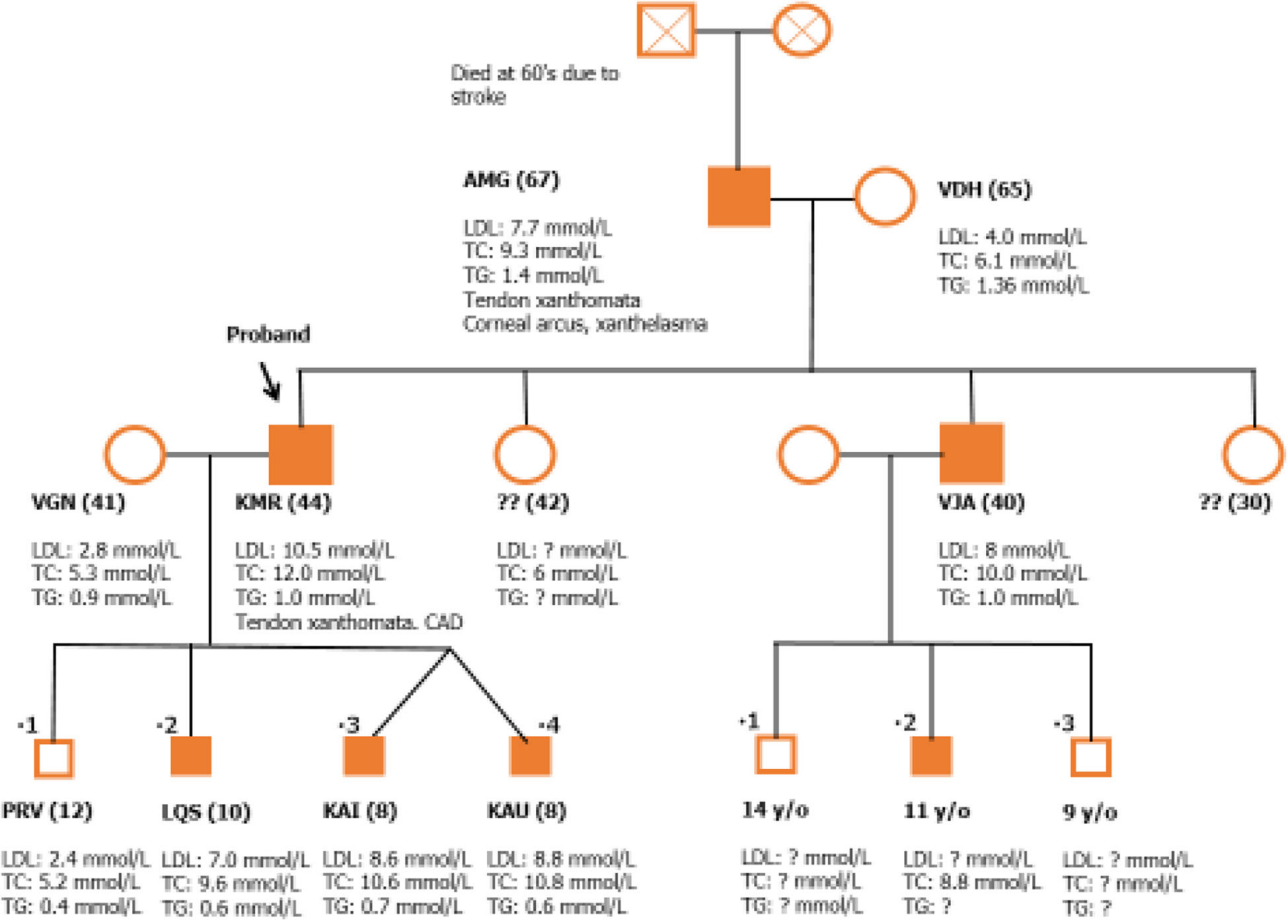
Family tree

Their liver function test, renal profile and thyroid function test were normal. They have no history of any cardiovascular complications. Screening of the other family members revealed that, their older brother (10-year-old) was also diagnosed as probable FH according to the DLCC criteria with TC of 10.8 mmol/L and LDL-C of 7.0 mmol/L. Both their 43-year-old father (index case) and 63-year-old paternal grandfather were also diagnosed with FH and they had premature CAD with presence of tendon xanthomata and corneal arcus. Their mother and the eldest brother, who is 12 years old were healthy with normal lipid profile. The twin have no history of any cardiovascular events. Both of them were clinically healthy. Advices were given for a low cholesterol diet, appropriate caloric intake and regular physical activity. Pharmacological treatment has not been initiated for the twins and their affected 10-year-old brother due to parental refusal in view of their young age. However, they are monitored closely under our care in a Specialist Lipid and Coronary Risk Prevention Clinic of a teaching hospital. The family tree is illustrated in Fig. 1.

In view of the strong family history of hypercholesterolemia and premature CAD**,** family cascade screening and genetic analyses were perfomed for the index case, affected and unaffected family members to detect for the pathogenic FH mutations following informed consent that were collected.

Blood samples were acquired from the proband and the other six family members including, the mother, the twins, two older brothers and the paternal grandfather. DNA was extracted from whole blood by using QIAamp Blood Mini Kit (Qiagen) according to standard manufacturer’s protocol. Extracted DNA was checked using agarose gel electrophoresis, followed by quantitation using Qubit fluorometer with dsDNA HS (High Sensitivity) Assay Kit (Thermo Fisher Scientific).

Targeted next-generation sequencing (TNGS) was implemented for comprehensive genetic analysis for the known and novel mutations in hot spots within exons and exon–intron boundaries of *LDLR* (39 amplicons), *APOB100* (106 amplicons), *PCSK9* (32 amplicons), *ABCG5* (22 amplicons), *ABCG8* (20 amplicons), *APOE* (8 amplicons) and *LDLRAP1* (23 amplicons). Average amplicon length is 242 bp.

Fifty nanogram of genomic DNA per individual were used for library preparation (TruSeq Custom Amplicon, Illumina). Next-generation sequencing kit (MiSeq v3, Illumina) was used according to manufacturers’ protocols to prepare the library and the samples were sequenced on a MiSeq sequencer (MiSeq sequencer, Illumina). Amplification was considered passed the QC if 95% of the nucleotides has ≥20X coverage.

Genomic sequences were mapped to GRCh37 hg19 human reference assembly using TruSeq Amplicon V3.00 proprietary cloud-based software. Variant calling was performed using Somatic Variant Caller (Illumina) and generated into VCF format. BaseSpace Variant Interpreter (Illumina) was used to annotate the variants. Variants with minor allele frequency of ≤0.02 (ExAC browser and 1000 Genome) were considered as mutants. All mutations were checked for pathogenicity by searching in ClinVar. In silico prediction of pathogenicity in exonic mutations were interpreted using Mutation Taster and Polypophen2, while intronic mutations (≤10 bp away from exon) were checked for their effect on splicing by using Human Splicing Finder and NetGene 2.

Pedigree of the twins showed a vertical transmission of the hypercholesterolemia from the father to the twin suggesting an autosomal dominant mode of inheritance. The proband (the father who was clinically diagnosed as definite FH by the DLCC) was identified to have one heterozygous missense mutation in *LDLR* gene (c.530C > T, p.Ser177Leu) that is located at exon 4 of this gene. This heterozygous mutation was also found in the older son (clinically diagnosed as probable FH by the DLCC) and grandfather (clinically diagnosed as definite FH by the DLCC) (Table 1). The clinically diagnosed younger twin pair siblings (both are clinically diagnosed as definite FH by the DLCC) has the same heterozygous *LDLR* mutation that is reported in the proband (530C > T, p.Ser177Leu). This missense mutation that was identified in this family was observed to co-segregate with the hypercholesterolaemia and has already been reported as probably damaging by Polyphen2 and deleterious by SIFT. Another missense variant was also reported in this twin pair, c.55G > C (p.Asp19His) which is located in exon 1 of the *ABCG8* gene and it was reported to be possibly damaging by Polyphen2 and SIFT. The mother and the other older brother who are normolipidemic were found to have heterozygous intronic variant (c.1060 + 7 T > C) that is located in intron 7 of the *LDLR* gene and was found to be a benign variant. Additionally, both of them were also found to be carriers for c.55G > C(p.Asp19His) variant in the *ABCG8* gene.

There was no nonsense or frameshift mutations that were observed in the *LDLR* gene sequence. The analysis of the samples showed no other pathogenic variants in the known FH genes (*APO B-100* and *PCSK9* genes), which could contribute to the phenotype of this family. Table 2 represents a summary of the gene variants characteristics that were reported in this family.

**Table 2:** Summary of genes and their variants which are identified by TNGS

Gene	dbSNP ID	Chromosomal location	Exon (intron)	DNA Sequence	Variant name
*LDLR*	rs121908026	Chr19:11216112	Exon 4	Reference: AGATGGCT**C**GGATGAGT Variant: AGATGGCT**T**GGATGAGT	Missense variant NM_000527.4:c.530C > T p.Ser177Leu
*LDLR*	rs2738442	Chr 19:11221454	Intron 7	Reference: GTGATT**T**CCGGGTGGGAC Variant: GTGATT**C**CCGGGTGGGAC	Intronic variant NM_000527.4: c.1060 + 7 T > C
*ABCG8*	rs11887534	Chr 2:44066247	Exon 1	Reference: CTCCCCAG**C**ATACCTCG Variant: CTCCCCAG**G**ATACCTCG	missense variant NM_022437.2:c.55G > C p.Asp19His

## Discussion and conclusion

This report highlights the importance of screening to identify FH in young children especially within family members diagnosed with FH. The 2016 ESC/EAS Guidelines for the Management of Dyslipidaemias outlines that family cascade screening is recommended when an index case of FH is being identified [23]. In children, the guideline also recommends testing from 5 years of age and even earlier if homozygous FH is highly suspicious [23].

Awareness on this disorder is somehow lacking even among the clinicians [5]. Early detection will allow immediate lipid-lowering medications to be commenced to reduce the risk of progression to CAD. Children diagnosed with FH require commencement of statin treatment as early as 8 to 10 years old [23]. Treatment with statin should be started with low doses and then increased to achieve the treatment goals. The goal in children above 10 years of age is LCL-c < 3.5 mmol/L whereas a level of at least a 50% reduction in the LDL-c level for younger children [23]. The concept of cumulative LDL-c burden highlights the importance of early commencement of treatment. For an individual with heterozygous FH, this LDL-c burden is reached by the age of 35 years if untreated, however this can be delayed to the age of 48 years if the treatment is started by the age of 18 years old and can be more delayed up to 53 years old with earlier treatment by the age of 10 years [5]. Therefore, early initiation of treatment is vital to reduce the risk of progression of atherosclerosis in later life.

Managing FH in young children is indeed challenging as evident in our case. Despite multiple counseling explaining the benefits of statin therapy which outweigh the risks, parents are still very reluctant to start treatment. Dietary and lifestyle modification alone are not sufficient and statins are still the cornerstone of FH management [22]. The thought of being dependent on medication lifelong possibly is the main reason behind their refusal. Furthermore, management challenges also include mental and socioeconomic factors particularly in the economically under-privileged countries in Asia. Treatment of psychosocial risk factors can counteract psychosocial stress, depression and anxiety faced by patients and their family members, thus facilitating behavioral change and improving quality of life and future prognosis [24]. Disease burden is not only faced by the affected family members but also the unaffected members caring for the patients.

The clinical diagnosis of FH is recommended to be confirmed by DNA analysis [23]. In view of the heterogeneous nature of the Malaysian population, it is very essential to screen the suspected FH patients for *LDLR* gene via testing for the unique allelic variants that were previously described plus the novel variants in this gene comprising the promoter and coding regions, splice sites and splice branch points in order to identify the causative mutations among the suspected FH patients [24]. Our research strategy is performed by confirming the genetic diagnosis of FH among the index case and the family members using the recently developed TNGS method to identify FH related gene mutations. To the best of our knowledge, that this is the first study which apply TNGS technique to identify the causative gene mutations for FH among Malaysian population. Two heterozygous mutations were identified in the *LDLR* gene: p.Ser177Leu (c.530C > T) and c.1060 + 7 T > C mutations. For p.Ser177Leu that is located in exon 4, at the region that is encoding the fourth repeat in the ligand binding domain of this gene [25], cytosine at nucleotide 530 was replaced by thymidine, with a subsequent changing of serine to leucine amino acid at codon 177. This amino acid is part of the highly conserved SerAspGlu sequence that is identified in the ligand binding domain of this gene [26]. This substitution slows the transport of the protein to the cell surface and the defective receptors will not be able to bind to the LDL-c in the proper normal way [27].

To date, this is the first study that reports this heterozygous disease-causing mutation (p.Ser177Leu) among Malaysian FH patients. However, it was previously reported among Portuguese [28], Polish [29], Spanish [30] and Czech FH patients [31].

It is challenging to predict the biological effects of missense mutations satisfactorily [32], principally in genes such as the *LDLR* where the rate of neutral alleles relative to disruptive-missense alleles is high [33]. Thormaehlen et al. established the biological effect of missense variants in the *LDLR* by an in vitro study. His group demonstrated that p.Ser177Leu mutation, identified among Italian subjects with acute myocardial infarction was described as a “disruptive-missense” variant as it strongly inhibited the *LDLR* protein function, by decreasing LDL-uptake to about 6–31% of the wild type LDLR [34]. This p.Ser177Leu mutation was found in ClinVar database as a pathogenic mutation for FH. For this family it is shown to co-segregate with hypercholesterolaemia and for this reason it is considered as a disease-causing mutation.

A second variant, c.1060 + 7 T > C in the *LDLR* gene was also reported in this study. It is located in the 3′ splice region of intron 7 of the *LDLR* gene and this variant was previously reported among the Malaysian population by Al-khateeb et al [35] Such alteration in non-coding regions close to the intron exon junction may potentially affect the splicing however this variant was reported to be a benign by the Polyphen2 and SIFT. Such evidence recommends that this variant may not have a damaging effect on *LDLR* function.

Another missense variant p.Asp19His, that was reported in the twin pair (hypercholesterolemic), mother and older brother (normolipidemic), but not in the other family members. It is located in exon 1 of *ABCG8* gene and was reported to be as a susceptibility factor for gallstone disease among German [36] and Chinese population [37]. This variant was also found to be associated with the disease mentioned above among African American and Hispanic American ancestry additionally among Indian population [38–40]. Those reports are suggesting that such variant might be associated with a more successful transportation of cholesterol in the bile. It was reported as propably damaging in Clin Var database.

Mutations that cause sitosterolemia are very rare. Common sequence variants in *ABCG8* may alter sterol metabolism and may lead to inter-individual variation in the plasma concentrations of plant sterols [41]. This p.Asp19His variant was reported among Hispanic subjects and was significantly associated with lower concentrations of plasma sterol concentration, suggesting that it may alter the function of ABCG8 by increasing its transporter function [42]. An association of this variant with plasma cholesterol concentration could not be observed [43] and this may explain the normal cholesterol level in the mother and eldest brother who are carriers of this variant. Our study showed that the twins who had both mutations (in LDLR and ABCG8 genes) had a more severe clinical phenotype with a higher TC and LDL-c compared to their father, grandfather and hypercholesterolemic brother who only had *LDLR* mutation. We postulate that *ABCG8* gene variant on the top of *LDLR* mutation may enhance the severity of the clinical phenotype in terms of the hypercholesterolemia in the twin patients. This finding also suggests that if untreated, the twins may have worse lipid profile and clinical phenotype in their later life. A population based study with an appropriate sample size or a protein based study may be indicated to reveal the effect of this variant on the LDLR function.

Patients suffering from sitosterolemia have also been described to primarily develop xanthomata and even premature coronary atherosclerosis [16]. However, our twins did not manifest any sign of xanthomas. The absence of xanthomata was probably due to weak physical manifestation of the *ABCG8* where it is only present in heterozygous form in the twins. However, there was evidence that the presence of rare *ABCG5*/*G8* in excess, even without the presence of *LDLR*, can cause significance increase in LDL-c level and cholesterol hyperabsorption [44] which explains why the LDL-c in the twins were higher than in their father and grandfather who were absent of *ABCG8* variant.

Diagnosis of sitosterolemia remains very challenging in children. Pediatric patients may manifest planar, tuberous, or tuberoeruptive xanthomata. Affected children may also present with profound hypercholesterolemia that is responsive to dietary management [45]. Sitosterolemia can be easily misdiagnosed as FH because of the similarity in clinical diagnosis [46, 47]. However, differentiation of sitosterolemia from FH is very important, since conventional high-vegetable (plant) oil cholesterol-lowering diet is contraindicated among those sitosterolemic patients as dietary therapy is aimed at reducing the phytosterol accumulation [45]. Both *ABCG5* and *ABCG8* were demonstrated to inhibit the absorption of cholesterol and plant sterols from the diet by mediating the efflux of these sterols from enterocytes back into gut lumen, and by promoting efficient secretion of cholesterol and plant sterols from hepatocytes into the bile [48]. In view of the essential role of ABCG5/ABCG8 in cholesterol removal from the body, it seems that overexpression of ABCG5 and ABCG8 would prevent atherosclerosis while a defect should promote atherosclerosis [49]. A large cohort study of patients with heterozygous FH showed that the ABCG8 rs11887534 variant have an association with higher risk of coronary heart disease [50].

This is the first in the literature to report a case of heterozygous FH in monochorionic diamniotic twin with mutations in both *LDLR* and *ABCG8* gene. Miyagi et al. reported a dichorionic diamniotic twin with compound heterozygous *LDLR* mutation causing FH^42^. Rabacchi et al. on the other hand reported identical twins with compound heterozygous FH with two *LDLR* gene mutations [43].

In summary, a rare case of Asian monochorionic diamniotic identical twin, of Indian descent, with clinically diagnosed and molecularly confirmed heterozygous FH, due to *LDLR* and *ABCG8* gene mutations have been reported. This is the first study that identified the disease-causing mutation p.Ser177Leu in the *LDLR* gene among Malaysian FH patients, suggesting that this mutation does not only exist among Caucasian population. Another variant in the *ABCG8* gene, p.Asp19His, was also identified for the first time among Malaysian FH population.

Childhood FH may not present with the classical physical manifestations including the pathognomonic lipid stigmata as in adults. Therefore, childhood FH can possibly be better diagnosed early using a combination of both clinical criteria and molecular analyses. Furthermore, identification of index cases in childhood, may provide opportunities for reverse family cascade screening, allowing greater advantage of early detection and prevention of CAD in other family members. In addition, it is important to obtain an early diagnosis of childhood FH to prevent premature atherosclerosis and CAD.

## Abbreviations

APO B-100: Apolipoprotein B-100 gene
CAD: Coronary artery disease
FH: Familial hypercholesterolaemia
HeFH: Heterozygous familial hypercholesterolaemia
HoFH: Homozygous familial hypercholesterolaemia
LDL-c: Low-density lipoprotein cholesterol
LDLR: Low-density lipoprotein receptor
PCSK9: Proprotein convertase subtilisin/kexin 9
TC: Total cholesterol
